# Does sodium nitroprusside kill babies? A systematic review

**DOI:** 10.1590/S1516-31802007000200008

**Published:** 2007-03-04

**Authors:** Nelson Sass, Caroline Harumi Itamoto, Marina Pereira Silva, Maria Regina Torloni, Álvaro Nagib Atallah

**Keywords:** Nitroprusside, Fetal death, Fetal mortality, High-risk pregnancy, Hypertension, Nitroprussiato, Morte fetal, Mortalidade fetal, Gravidez de alto risco, Hipertensão

## Abstract

**OBJECTIVE:**

To determine whether sodium nitroprusside causes fetal death in pregnancies complicated with hypertension.

**DATA SOURCES:**

Medical Literature Analysis and Retrieval System Online (MEDLINE; 1996 to 2003), Excerpta Medica (EMBASE; 1970 to 2003), Web of Science/Institute for Scientific Information (ISI; 1945 to 2003), Literatura Latino-Americana e do Caribe em Ciências da Saúde (LILACS; 1982 to 2003) and the Cochrane Library.

**REVIEW METHODS:**

The medical subject headings used were "nitroprusside and pregnancy", "hypertension or eclampsia or preeclampsia" and "nitroprusside and pregnancy and hypertensive emergencies". The search was limited to humans and female gender, in all fields, publication types, languages and subsets. Articles were also identified by reviewing the references of articles and textbooks on hypertension and pregnancy.

**RESULTS:**

The search located nine studies. The sum of all the publications yielded a total of 22 patients and 24 exposed fetuses (two pairs of twins). There were no randomized clinical trials and no prospective cohorts. All of the studies were observational in nature.

**CONCLUSIONS:**

At present, there is insufficient evidence for definitive conclusions about any direct association between sodium nitroprusside use and fetal demise.

## RESUMO

**OBJETIVOS:**

Resolver a questão: o nitroprussiato de sódio causa morte fetal em gestações complicadas por hipertensão?

**FONTES DE INFORMAÇÃO:**

Medical Literature Analysis and Retrival System Online, MEDLINE (1996 a 2003), Excerpta Medica, EMBASE (1970 to 2003), Web of Science - Institute for Scientific Information, ISI (1945 a 2003), Literatura Latino-Americana e do Caribe em Ciências da Saúde, LILACS (1982 a 2003) e a Cochrane Library.

**MÉTODO DE REVISÃO:**

Os descritores usados foram "nitroprussiato e gravidez", "hipertensão ou eclâmpsia ou pré-eclâmpsia", "nitroprussiato e gravidez e emergências hipertensivas" limitada a humanos e mulheres, em todos os campos, tipos de publicação, línguas e subgrupos. Foram também identificados artigos através das referências das publicações e de livros sobre hipertensão e gravidez.

**RESULTADOS:**

A revisão identificou nove publicações. A soma de todas elas descreveu um total de 22 mulheres e 24 fetos (duas gestações gemelares) expostos ao nitroprussiato. Não foram localizados ensaios clínicos randomizados ou coortes prospectivas. Todos os estudos eram do tipo descritivo.

**CONCLUSÕES:**

No momento, não existem evidências suficientes para se chegar à conclusão definitiva de que exista uma associação direta entre o uso de nitroprussiato de sódio e morte fetal.

## INTRODUCTION

Hypertensive disorders are a leading cause of maternal and perinatal morbidity and mortality worldwide. A large population-based study^1^ quantified the risk of fetal growth restriction and stillbirths in pregnancies with hypertensive disorders. After controlling for potential confounding factors, the risk of fetal death was 1.4 times greater (95% confidence interval, CI: 1.1-1.8;p = 0.02) in women with any hypertensive disorder and 3.2 times greater (95% CI: 1.9-5.4; p < 0.001) in those with pre-existing hypertension, in comparison with normotensive women.

Hypertensive emergencies are frequent, and are associated with substantial maternal and perinatal morbidity and mortality. Although hypertension is a common denominator, the pathogenesis, clinical features and clinical course are variable and somewhat distinct.^2,3^ Table 1 presents the possible clinical manifestations and consequences of hypertensive crises during pregnancy. Once diastolic blood pressure reaches 110 mmHg, there is a general consensus that the patient should receive antihypertensive drugs in a hospital. The medications used in this situation may affect the fetus indirectly, through their effect on uteroplacental perfusion, or directly, after crossing the placenta and entering the fetal circulation.^4^

**Table 1:** Clinical manifestations and consequences of hypertensive emergencies in pregnancy

Acute aortic dissection
Acute renal failure
Angina pectoris
Eclampsia
Hypertensive encephalopathy
Microangiopathic hemolytic anemia
Myocardial infarction
Pulmonary edema

Adapted from Varon and Marik (2000).^2^

So far, there has not been any clear evidence indicating that any specific antihypertensive agent is superior. Hydralazine is a popular first choice, but clinical experience with nifedipine and labetalol is mounting. According to a recent systematic review,^4^ except for diazoxide and ketanserin (which should be avoided), the choice of which specific antihypertensive drug to use should be based mainly on the experience of the individual clinician.

There is a need to consider sodium nitroprusside (SNP) in this respect. There seems to be a general impression that SNP is lethal to the fetus, although there may be some doubt about this. On the other hand, there is uncertainty regarding the strength of the evidence for banning its use in clinical practice. The aim of the present study was to review the available clinical data relating to the use of SNP in pregnant women with hypertensive crises.

## METHODS

Searches to locate articles relating to the clinical use of SNP during pregnancy were conducted in Medical Literature Analysis and Retrieval System Online (MEDLINE; 1996 to 2003), Excerpta Medica (EMBASE; 1970 to 2003), Web of Science/Institute for Scientific Information (ISI; 1945 to 2003), Literatura Latino-Americana e do Caribe em Ciências da Saúde (LILACS; 1982 to 2003) and the Cochrane Library. The medical subject hea-dings used were "nitroprusside and pregnancy", "hypertension or eclampsia or preeclampsia" and "nitroprusside and pregnancy and hypertensive emergencies". The search was limited to humans and female gender, in all fields, publication types, languages and subsets. Articles were also identified by reviewing the references of articles and textbooks on hypertension and pregnancy.

## RESULTS

The search located nine studies,^5-13^ which are presented in Table 2. The sum of all the publications yielded a total of 22 patients and 24 exposed fetuses (two pairs of twins). There were no randomized clinical trials and no prospective cohorts. All of the studies were observational in nature.

**Table 2:** Use of sodium nitroprusside (SNP) in pregnancy: case reports

Author	gestational age	clinical findings	drugs before SNP	no. of fetuses	total SNP dose used	duration of SNP use	fetal outcome
Paull, 1975^5^	nd	severe PIH,	diazoxide	1	nd	nd	liveborn
		renal failure,					
		coagulopathy					
Donkin et al., 1978^6^	7 months	intracranial	diazepam	1	12.5 mg	nd	liveborn
		aneurysm	atropine				3200 g
Rigg and McDonogh, 1981^7^	20 weeks	intracranial	propranolol	1	90.0 mg	96 hours	liveborn
		aneurysm	methyldopa				3570 g
	20 weeks	intracranial	nd	1	60.0 mg	nd	liveborn
		aneurysm					3205 g
Stempel et al., 1982^8^	38 weeks	severe PIH	magnesium sulfate hydralazine	2	0.483 mg/kg	42.9 hours	both liveborn 2340 g, 2670 g
	26 weeks	severe PIH	magnesium sulfate	1	0.219 mg/kg	38 hours	liveborn 730 g
	33 weeks	chronic hypertension	hydralazine furosemide	1	nd	38 hours	liveborn 1927 g
	31 weeks	severe PIH, diabetes	magnesium sulfate hydralazine	1	0.525 mg/kg	50 hours	liveborn 1700 g
Willoughby, 1984^9^	16 weeks	intracranial aneurysm	nd	1	25.0 mg	nd	liveborn 3340 g
Shoemaker and Meyer, 1984^10^	24 weeks	severe PIH pulmonary edema	magnesium sulfate hydralazine	1	3.5 mg/kg	15 hours	stillborn 478 g
Baker, 1990^11^	41 weeks	severe PIH	labetalol phenytoin	1	nd	36 hours	liveborn 3630 g
	28 weeks	severe PIH	hydralazine phenytoin	1	nd	2 hours	liveborn 830 g
	34 weeks	severe PIH	labetalol hydralazine	2	nd	12 hours	both liveborn 2520 g, 2250 g
	35 weeks	severe PIH	labetalol hydralazine	1	nd	2 hours	liveborn 2460 g
	28 weeks	severe PIH	labetalol hydralazine phenytoin	1	nd	1 hour	liveborn 1324 g
	36 weeks	severe PIH	captopril nifedipine	1	nd	4 hours	liveborn 2400 g
	34 weeks	severe PIH	hydralazine	1	nd	3 hours	liveborn 1770 g
	28 weeks	severe PIH	labetalol nifedipine	1	nd	6 hours	liveborn 995 g
Mendonça et al., 1994^12^	nd	severe PIH	nd	1	nd	nd	stillborn
	nd	severe PIH	nd	1	nd	nd	stillborn
	nd	severe PIH	nd	1	nd	nd	stillborn
Nascimento and Vale, 1998^13^	34 weeks	severe PIH pulmonary edema	hydralazine nifedipine furosemide morphine	1	7.0 μg/kg/min	3 hours	stillborn

nd = not described; PIH = pregnancy-induced hypertension.

Eighteen women received sodium nitroprusside for severe hypertension and four (in case reports) were being treated for intracranial aneurysms. All the hypertensive patients had previously received at least one other antihypertensive drug.

Only 6 publications mentioned the SNP infusion rate and it was different in all cases. The duration of SNP use ranged from 1 to 96 hours, and was not mentioned in 7 cases. The gestational age ranged from 16 to 41 weeks and fetal weight from 478 g to 3650 g. For 4 out of 5 stillbirths, there was no information regarding gestational age or fetal weight. All stillbirths occurred among patients with severe hypertension. Many different antihypertensive agents had been used before SNP, and hydralazine was the most frequent. All patients other than those with cerebral aneurysm had other significant clinical problems relating to severe and persistent hypertension.

## DISCUSSION

Currently, the most popular drug for treating severe hypertension in pregnancy is hydralazine, probably due to its long record of safety and efficacy.^14,15^ Labetalol and nifedipine have similar hemodynamic effects and are also widely used.^16,17^ Although some reports have indicated that sodium nitroprusside is also effective and safe when infused for short periods and at low doses,^18^ obstetricians rarely prescribe nitroprusside and the experience of its use during pregnancy is very limited.

Sodium nitroprusside is an ultrafast antihypertensive agent that produces arterial and venous vasodilatation, thereby decreasing both preload and afterload.^19^ Because of this effect, SNP is a good choice for treating pulmonary edema, even in pregnancy.^20^ It has an action time of one to two minutes and a plasma half-life of three to four minutes.^21,22^ If the infusion is stopped, the blood pressure returns to the pretreatment levels within one to ten minutes. Sodium nitroprusside is more potent than hydralazine for reducing blood pressure^23^ and, because of the decrease in systemic pressure, it significantly reduces blood flow to the kidneys, uterus and placenta.^24,25^ In pregnant and non-pregnant women, SNP also produces more intense uterine artery dilatation than does hydralazine. And while the placental veins of preeclamptic and normotensive women of similar gestational age are equally sensitive to SNP, the effect is greater in preterm pregnancies.^26^ The results from animal studies support the idea that SNP use for hypertensive emergencies deserves reappraisal and its use during general endotracheal anesthesia merits further investigation.^27^ Almost all publications on SNP mention the possible fetal risk due to cyanide toxicity, which was initially demonstrated in animal studies. One of these studies, which was designed to determine the maternal and fetal hemodynamic effects of SNP in normal and hypertensive animal models,^28^ demonstrated that the drug crosses the placenta and that the fetal concentration of cyanide is related to maternal SNP levels. Another experiment reported that five out of eight pregnant ewes exhibited tachyphylaxis to SNP, followed by fetal deaths.^21^ Nevertheless, these authors commented that SNP could be a useful drug for short-term treatment of life-threatening hypertension in patients with severe preeclampsia. They recommended that the mother and fetus should be carefully monitored during the infusion, especially when using doses greater than 0.5 µg/kg per hour.^29^

SNP is metabolized into cyanogens and converted into thiocyanate by thiosulfate sulfurtransferase.^30^ Nitroprusside contains 44% cyanide by weight, and this is released non-enzymatically from the drug. The amount of cyanide produced depends on the dose administered, and it is metabolized in the liver to thiocyanate, a substance that is one hundred times less toxic than cyanide. Cyanide removal also requires adequate renal function and bioavailability of thiosulfate.^31^ SNP may cause cytotoxicity through the release of nitric oxide, thus leading to lipid peroxidation^32,33^ and the release of cyanide, which interferes with cell respiration.^34,35^ SNP infusion rates greater than 4 µg/kg/min for as little as two to three hours may lead to cyanide levels that are within the toxic range and, therefore, an infusion of thiosulfate should be used. Doses greater than 10 µg/kg/min produce cyanide at a far greater rate than human beings can detoxify it.^30^ Table 3 presents the recommendations for clinical use.^36^

**Table 3:** Sodium nitroprusside: doses recommended for clinical use

Initial dose	0.3-1.0 µg/kg/minute
Mean dose	3.0 µg/kg/minute
Maximum dose	8.0 µg/kg/minute
If long-term use is necessary (over periods of hours or days)	limited to 2.5 µg/kg/minute
Limits of cyanide tolerability	blood: 100.0 µg/100 ml plasma: 8.0 µg/100 ml

Adapted from DEF (2002).^37^

Considering the available evidence from animal and human studies, we believe it is impossible to conclude that SNP itself was directly and entirely responsible for the fetal deaths presented. In fact, data from the publications with the largest numbers of severely hypertensive patients suggest that maternal and fetal toxicity may not be a serious concern when SNP is used for short periods in emergency situations.^8,11^ On the other hand, the cases of fetal deaths attributed to SNP are poorly documented.^10,12,13^ First, many authors failed to report important information such as dosage and duration of use of SNP, as well as the cyanide concentration in maternal blood or fetal tissue. Second, in all the cases reported (except study number 12), SNP was never the only drug involved. It was always prescribed for severely hypertensive patients after one or more antihypertensive drugs had failed to reduce blood pressure, thus making it difficult to reach the conclusion that SNP was directly responsible for fetal demise. The risk of perinatal death during hypertensive emergencies is considerable. As pointed out by a systematic review,^4^ the fetal death rate among patients treated with hydralazine was 5/122 (4.1%), with labetalol 1/76 (1.3%) and nifedipine 6/75 (8.0%). Among the 18 patients with severe hypertension who used SNP there was a high rate of stillbirths (5/18 or 27.8%), but it is questionable whether, based on such a small sample, it possible to conclude that the risk of fetal death was significantly higher among patients who received SNP.

These results may be due in part to differences in the clinical settings within which each drug was used. SNP was always prescribed for the most severe cases, and after one or more antihypertensive drugs had failed to reduce blood pressure. Therefore, babies whose mothers received SNP were more likely to be severely hypoxic because of their inherent clinical condition. It needs to be considered whether the result would have been the same if SNP had been used as the first choice. It may have been the case that placental function deteriorated and fetal conditions worsened between patient admission and the time when SNP was used. Chance and confounding factors might also be implicated in these results, which were observed in a very number of patients. It is impossible to answer such questions at the moment.

On the basis of the available data, there is insufficient evidence for any reliable recommendation about the use of SNP for severe hypertension in pregnancy. There is insufficient evidence for definitive conclusions about any direct association between SNP use and fetal demise.
